# A Cross-Lagged Study of Helicopter Parenting, Parent–Child Conflict, and Adolescents’ Short Video Addiction: The Moderating Role of Trait Autonomy

**DOI:** 10.3390/bs16050729

**Published:** 2026-05-09

**Authors:** Hengzhe Wang, Xingchao Wang, Fanwei Meng, Haiying Wang

**Affiliations:** 1 School of Psychology, Northeast Normal University, Changchun 130024, China; wanghengzhe669@nenu.edu.cn; 2 School of Educational Science, Shanxi University, No. 92 Wucheng Road, Taiyuan 030006, China; wangxch@sxu.edu.cn (X.W.); mfw199808@163.com (F.M.)

**Keywords:** helicopter parenting, short video addiction, parent–child conflict, trait autonomy

## Abstract

Short video addiction has become an increasingly important problem among adolescents, yet its family antecedents remain understudied. The present study used a cross-lagged design to examine relations among adolescent-reported helicopter parenting, parent–child conflict, and short video addiction, as well as the moderating role of trait autonomy. Cross-lagged data were collected from Chinese adolescents (*N* = 1051; mean age at baseline = 16.42 years; standard deviation = 0.83). Results showed that helicopter parenting at Time 1 (T1) did not predict short video addiction at Time 2 (T2), whereas short video addiction at T1 positively predicted helicopter parenting at T2. Bidirectional associations were found between helicopter parenting and parent–child conflict, and between parent–child conflict and short video addiction. In addition, a small indirect association from T1 helicopter parenting to T2 short video addiction through parent–child conflict was observed. Compared with adolescents with high trait autonomy, helicopter parenting, parent–child conflict, and short video addiction showed greater stability from T1 to T2 among adolescents with low trait autonomy. These findings underscore the importance of considering both family relationship processes and adolescents’ self-regulatory characteristics in interventions for short video addiction.

## 1. Introduction

Adolescents are at the forefront of digital technology use (Rodríguez-de-Dios et al., 2018). With the rapid development of the internet, short videos have become one of the main forms of entertainment for adolescents (Weimann & Masri, 2023). Among nearly 200 million adolescent internet users in China, 67.9% use short video platforms (China Internet Network Information Center, 2024). Adolescents’ openness to novel digital experiences makes them particularly active users of short-form video platforms (Smith & Short, 2022). Although short videos provide entertainment and social connection, their highly engaging content, immersive design, and personalized recommendation algorithms also increase the risk of excessive and poorly controlled use (Qu et al., 2024). Short video addiction refers to problematic short video use characterized by addiction-like symptoms, rather than a formal clinical diagnosis (H. Wang & Lei, 2022). This construct captures a maladaptive pattern of short video use characterized by impaired control, excessive preoccupation, functional impairment, and use for emotional escape (H. Wang & Lei, 2022). In addition, short video addiction has significant adverse effects on adolescents’ psychological and behavioral health, such as depression and sleep disorders (Swathi & Devakumar, 2020). Thus, given the growing popularity of short videos and the negative impact of short video addiction on adolescent development, it is important to identify factors that contribute to its development.

As a result of physical, cognitive, and social maturation, adolescents have a greater need for autonomy and a desire for equal status in family interactions (Lila et al., 2020). Adolescents are more vulnerable to problematic short video use when parents do not respect their autonomy (Swathi & Devakumar, 2020). Helicopter parenting, a parenting style characterized by low autonomy granting and excessive control, has been linked to poorer adolescent adjustment and problematic internet use (Y. Wang et al., 2025). However, the association between helicopter parenting and adolescents’ short video addiction remains unclear. Moreover, helicopter parenting may create unequal parent–child interactions and provide a context for conflict (Y. Wang & Hawk, 2023). Adolescents in conflict-ridden family environments are more likely to engage in problematic short video use (J. Wang et al., 2023). Despite these insights, little is known about whether parent–child conflict serves as an intervening pathway in the association between helicopter parenting and short video addiction. Thus, within the transactional model of development and from a family systems perspective, the current study used a cross-lagged design to examine associations among helicopter parenting, parent–child conflict, and short video addiction, and further explored whether these associations were moderated by trait autonomy.

### 1.1. Helicopter Parenting and Short Video Addiction

Helicopter parenting is defined as a pattern of developmentally inappropriate overinvolvement by parents who “hover” over adolescents to ensure that they are successful and safe from harm (Ching et al., 2023; Turner et al., 2020). It is characterized by high parental warmth and support but also by high control and low autonomy granting (La Rosa et al., 2025; Padilla-Walker & Nelson, 2012). The transactional model of development posits that parenting practices and adolescents’ developmental outcomes may influence each other over time (Sameroff, 2009). As an intrusive form of parental involvement, helicopter parenting may create a controlling family context (Padilla-Walker & Nelson, 2012). In such contexts, offspring use the internet as a way to cope with or escape from overinvolved parenting behaviors (Love et al., 2022). Given that short video content is easily accessible, entertaining, and relaxing (Smith & Short, 2022), it is reasonable to speculate that adolescents experiencing helicopter parenting may use short videos as a way to escape overcontrol, which may increase their likelihood of short video addiction. In addition, although direct evidence from adolescent samples remains limited, previous empirical research found a positive association between helicopter parenting and short video addiction among college freshmen (L. Li et al., 2024).

Simultaneously, adolescents are also active shapers of their social environments, including their interactions with parents (Lila et al., 2020). Based on the transactional model of development, adolescents’ problem behaviors trigger ineffective parenting strategies (Steeger & Gondoli, 2013). Thus, adolescents who spend excessive time on short videos may experience greater parental restriction and perceive it as increased helicopter parenting. For instance, one longitudinal study found that higher levels of online game addiction among children were associated with higher levels of parents’ internet-specific reactive restrictions (Koning et al., 2018). However, to our knowledge, empirical research has rarely examined whether adolescents’ short video addiction predicts later helicopter parenting. Therefore, the current cross-lagged study may provide preliminary evidence regarding whether adolescents’ short video addiction is prospectively associated with later helicopter parenting.

### 1.2. Helicopter Parenting and Parent–Child Conflict

Parent–child conflict refers to a state of tension or antagonism between parents and children arising from differences in viewpoints, expectations, and behaviors (Smetana et al., 1991). From the perspective of behavioral family systems, maladaptive parenting disrupts the balance of family functioning and increases the likelihood of parent–child conflict (Robin & Foster, 2002). Helicopter parenting, as a form of enmeshment, disrupts the balance between separation and connectedness within the family system, thereby triggering conflict (McCoy et al., 2024; Segrin et al., 2012). In addition, helicopter parenting is inconsistent with adolescents’ changing and decreasing dependency needs, which increase the occurrence of parent–child conflict (Leung, 2021). This association may also emerge in the short term. For example, a short-term longitudinal study found that maternal helicopter parenting positively predicted adolescents’ perceived conflict two weeks later (Y. Wang et al., 2025). Taken together, these findings suggest that examining relations between helicopter parenting and parent–child conflict over a short time frame may help explore proximal developmental processes.

The pathway from parent–child conflict to helicopter parenting is also plausible. According to the transactional model of development, parents’ parenting behaviors may also be influenced by other levels of the developmental system, such as parent–child conflict (Sameroff, 2009; Sun et al., 2021). Conflicts between adolescents and parents over daily life issues may elicit subsequent parental control behaviors. For example, prior longitudinal research has shown that parent–child conflict positively predicts later parental psychological control (Steeger & Gondoli, 2013; Sun et al., 2021). However, evidence remains limited regarding whether parent–child conflict influences helicopter parenting. Only one study found that, although the effect size was small, mother–child conflict positively predicted maternal overparenting at the next time point (Leung, 2021). Therefore, it is necessary to examine whether parent–child conflict predicts subsequent helicopter parenting and to further investigate whether bidirectional associations exist between the two.

### 1.3. Parent–Child Conflict and Short Video Addiction

Families are important contexts in which adolescents use short video apps (Lei et al., 2024). According to the transactional model of development, dysfunctional parent–child relationships increase adolescents’ risk of deviant behaviors (Sameroff, 2009). In other words, adolescents experiencing parent–child conflict are likely to become more dependent on short videos. Parent–child conflict not only hinders adolescents’ growing need for individuation but also undermines their emotional security (Martin et al., 2019). Therefore, adolescents are likely to rely on short videos to meet social and psychological needs that are unmet within the family context (Lei et al., 2024; J. Yang et al., 2022). Although direct evidence remains limited, indirect evidence suggests that parent–child conflict is positively associated with adolescents’ problematic internet use (Özaslan et al., 2022). In addition, parent–child conflict is a major source of stress during adolescents’ developmental stages (Xu et al., 2021). Short video platforms provide adolescents with a virtual environment in which they can immerse themselves and temporarily distance themselves from real-life pressures (J. Yang et al., 2022). Thus, it is reasonable to speculate that parent–child conflict is likely to be a risk factor for adolescents’ short video addiction.

The reverse pathway from short video addiction to parent–child conflict is also possible. The transactional model of development suggests that adolescents’ problem behaviors may also influence their relationships with parents (Sameroff, 2009). Adolescents’ short video addiction may disrupt family interactions and displace shared parent–child time, thereby increasing the risk of parent–child conflict. For example, problematic internet use, as a maladaptive behavior that presents noncompliance with parental instructions and family norms, may strongly trigger parent–adolescent conflict (Liu et al., 2024). In addition, the negative consequences associated with short video addiction may become a concern for parents (J. Wang et al., 2023). Such concerns may lead to disagreements between parents and adolescents, thereby increasing parent–child conflict (Liu et al., 2024). Previous research also provides indirect support for this view, showing that parent–adolescent conflicts over internet use are largely due to disagreements about usage (X. Yang & Zhang, 2021).

### 1.4. The Indirect Effect of Parent–Child Conflict

Parent–child conflict is an important relational experience between parents and adolescents and is viewed as reflecting the “storm and stress” characterization of adolescence (Weymouth et al., 2016). According to behavioral family systems theory, maladaptive parenting contributes to parent–child conflict, which in turn increases the risk of adolescents’ problem behaviors (Robin & Foster, 2002). From this perspective, helicopter parenting may indirectly influence adolescents’ short video addiction through parent–child conflict. A study indirectly supports this view, showing that mother–adolescent conflict mediates the association between maternal psychological control and adolescents’ problem behaviors (Steeger & Gondoli, 2013). Thus, parent–child conflict, as a key component of relational imbalance, may serve as a bridge linking helicopter parenting to adolescents’ short video addiction.

### 1.5. The Moderating Role of Trait Autonomy

Autonomy is an important developmental theme during adolescence (Deci & Ryan, 2012). According to self-determination theory, autonomy reflects self-regulation and volitional functioning, which contribute to psychological need satisfaction and adaptive development (Ryan & Deci, 2017). Although autonomy can be shaped by the social environment, adolescents also differ in relatively stable autonomous functioning, often referred to as trait autonomy or dispositional autonomy (Weinstein et al., 2012). Individuals with high trait autonomy may be better able to cope with controlling elements in the environment, such as overparenting, and may be less likely to experience such parental involvement as persistently intrusive (Jiao & Segrin, 2023; Ryan & Deci, 2017). They also tend to maintain close relationships with others and may be less prone to interpersonal conflict (Deci & Ryan, 2012; Ryan & Deci, 2017). In addition, adolescents with high trait autonomy may be less likely to develop short video addiction. For example, compared with adolescents with low trait autonomy, those with high trait autonomy are less likely to engage in deviant behaviors (Weinstein et al., 2019). Taken together, trait autonomy may be particularly relevant to the short-term stability of helicopter parenting, parent–child conflict, and short video addiction.

## 2. The Current Study

The present study contributes to the literature in three ways. First, the current study used a cross-lagged design to examine helicopter parenting, parent–child conflict, and adolescents’ short video addiction, thereby extending research on how family factors and adolescents’ short video addiction are linked over time. Second, this study moved beyond direct associations by examining whether parent–child conflict served as a potential intervening pathway linking helicopter parenting with adolescents’ short video addiction. Third, the present study further investigated whether the temporal stability of these family and behavioral constructs varied by adolescents’ trait autonomy. Specifically, based on the transactional model of development, bidirectional associations were hypothesized between helicopter parenting and short video addiction (Hypothesis 1a), between helicopter parenting and parent–child conflict (Hypothesis 1b), and between parent–child conflict and short video addiction (Hypothesis 1c). From a family systems perspective, helicopter parenting at T1 was expected to show an indirect association with short video addiction at T2 through parent–child conflict (Hypothesis 2). In addition, according to self-determination theory, trait autonomy would moderate the autoregressive paths from T1 to T2 of helicopter parenting, parent–child conflict, and short video addiction (Hypothesis 3).

## 3. Method

### 3.1. Participants

The data were from a cross-lagged study project about adolescents’ mental health. The three-month interval was selected to capture short-term cross-lagged associations, particularly as parent–child conflict and short video addiction may fluctuate over relatively short periods. An a priori power analysis was conducted using G*Power version 3.1.9.7 (Faul et al., 2007). Given that the cross-lagged paths in the two-wave CLPM can be viewed as regression paths predicting T2 variables from T1 variables and covariates, we used a regression-based approximation. Specifically, we selected the F-test option for linear multiple regression with a fixed model and R^2^ increase, using f^2^ = 0.02, α = 0.05, and power = 0.95. The number of tested predictors was 1, and the total number of predictors was 7, including the prior level of the outcome, the other two T1 study variables, and four covariates. Thus, the numerator degrees of freedom were 1 and the denominator degrees of freedom were N − 7 − 1. The analysis indicated that a minimum sample size of 652 was required, which was smaller than the final analytic sample of 990. This calculation was used as an approximate power analysis for individual cross-lagged paths rather than as a formal power analysis for the full structural equation model. For the present study, a total of 1051 students from two middle schools in Datong and Harbin, China, were initially invited to participate in the current study. After excluding students who did not provide valid baseline responses, 990 adolescents provided valid T1 data and were included in the FIML analytic sample. At T2, valid responses were obtained from 816 adolescents for helicopter parenting and parent–child conflict, 812 adolescents for short video addiction, and 814 adolescents for trait autonomy. However, the high- and low-trait-autonomy groups were formed using T1 trait autonomy scores only. Accordingly, although the number of valid cases varied slightly across T2 measures, missing data were handled using FIML within the SEM framework. The main reasons for attrition were transferring to another school or being absent from class during data collection. Difference test results showed no differences in scores for helicopter parenting (*M*_dropout_ = 2.35, *SD*_dropout_ = 0.50, *M*_stay_ = 2.51, *SD*_stay_ = 0.61; *t* = −0.73, *p* = 0.46), short video addiction (*M*_dropout_ = 2.83, *SD*_dropout_ = 0.31, *M*_stay_ = 2.38, *SD*_stay_ = 0.82; *t* = 0.94, *p* = 0.34), and trait autonomy (*M*_dropout_ = 2.90, *SD*_dropout_ = 0.49, *M*_stay_ = 2.90, *SD*_stay_ = 0.37; *t* = −0.01, *p* = 0.98) at T1, excepting parent–child conflict at T1 (*M*_dropout_ = 2.37, *SD*_dropout_ = 0.81, *M*_stay_ = 2.13, *SD*_stay_ = 0.79; *t* = 3.19, *p* = 0.007). Attrition analyses indicated that adolescents who dropped out after T1 reported significantly higher levels of parent–child conflict at baseline than those who remained in the study, *t* = 3.19, *p* = 0.007, suggesting selective attrition on a focal study variable. Sample characteristics for the educational level of participants’ mothers and fathers, per capita monthly family income, and areas at baseline are shown in Table 1.

### 3.2. Measures

Helicopter parenting. The seven-item Helicopter Parenting Scale developed by LeMoyne and Buchanan (2011) was used to assess adolescents’ perceived helicopter parenting. One sample item was “In life, my parents often help me with my problems.” It used a five-point scale ranging from 1 = strongly disagree to 5 = strongly agree. In the current study, CFA results indicated good model fit at T1 (*χ*^2^(9) = 20.14, RMSEA = 0.039, CFI = 0.98, TLI = 0.95, SRMR = 0.028). Model fit at T2 was generally acceptable (*χ*^2^(4) = 17.20, RMSEA = 0.064, CFI = 0.98, TLI = 0.89, SRMR = 0.019). Although the TLI at T2 was slightly below the conventional 0.90 criterion, the remaining fit indices were within acceptable ranges. Cronbach’s α values were 0.85 and 0.83 at T1 and T2. McDonald’s ω values were 0.85 and 0.83, respectively, at T1 and T2.

Parent–child conflict. Parent–child conflict was assessed using the 16-item Parent–Child Conflict Scale (Moos & Moos, 1976). The scale assesses adolescents’ conflicts with parents during the past month across eight domains, such as academic performance and homework, household chores, peer relationships, daily routines, and privacy-related issues. Adolescents rated both the frequency and intensity of conflict in each domain, resulting in 16 ratings. For example, they reported how often and how intensely they had conflicts with parents about academic performance and homework. Frequency was rated from 1 = never occurred to 5 = several times a day, and intensity was rated from 1 = never occurred to 5 = very intense. Following Treutler and Epkins (2003), frequency and intensity scores were multiplied and then averaged across domains, with higher scores indicating higher levels of parent–child conflict. This scale has shown good reliability and validity among Chinese adolescents (X. Li et al., 2014). In the current study, confirmatory factor analysis showed good model fit at T1, *χ*^2^(20) = 46.92, RMSEA = 0.041, CFI = 0.98, TLI = 0.97, SRMR = 0.024. The model also showed good fit at T2, *χ*^2^(20) = 72.17, RMSEA = 0.057, CFI = 0.97, TLI = 0.95, SRMR = 0.027. Cronbach’s α values were 0.93 and 0.94 at T1 and T2, respectively. McDonald’s ω values were 0.93 and 0.94 at T1 and T2, respectively.

Short video addiction. The Short Video Addiction Scale was adapted from Young’s (1998) Internet Addiction Scale. Following H. Wang and Lei (2022), the original items were revised by replacing “internet” with “short videos.” This adaptation was considered appropriate as the items assess core addiction-related symptoms, such as loss of control, excessive use, and negative functional consequences, which are also central to short video addiction. One sample item was “Are you actually spending more time swiping short videos than you planned?” It used a five-point scale ranging from 1 = strongly disagree to 5 = strongly agree. The higher scores indicated higher levels of short video addiction. This scale has shown good reliability and validity among Chinese adolescents (H. Wang & Lei, 2022). In the current study, CFA results indicated good model fit at T1 (*χ*^2^(14) = 36.477, CFI = 0.987, TLI = 0.975, RMSEA = 0.044, 90% CI [0.027, 0.062], SRMR = 0.022). The model showed acceptable fit at T2, *χ*^2^(14) = 81.891, CFI = 0.963, TLI = 0.927, RMSEA = 0.070, 90% CI [0.061, 0.094], SRMR = 0.038. Cronbach’s αs were 0.86 and 0.87, respectively, at T1 and T2. McDonald’s ω values were 0.86 and 0.87, respectively, at T1 and T2.

Trait autonomy. Trait autonomy was examined using the 15-item scale from the Index of Autonomous Functioning Scale (Weinstein et al., 2012). One sample item was “I strongly identify with the things that I do.” It used a five-point scale ranging from 1 = strongly disagree to 5 = strongly agree. Scores from all items were averaged with a higher score reflecting a higher level of trait autonomy. This scale has shown good reliability and validity among Chinese adolescents (Yu et al., 2021). In the current study, confirmatory factor analysis showed generally acceptable model fit at T1, *χ*^2^(87) = 245.56, RMSEA = 0.047, CFI = 0.91, TLI = 0.89, SRMR = 0.045. Cronbach’s α was 0.93 and McDonald’s ω was 0.91 at T1.

### 3.3. Procedure

All materials and procedures were approved by the Shanxi University Ethics Committee. Then, the corresponding author obtained informed consent from the participants, parents, and teachers. At each time point, participants were informed that their responses were confidential and that they could terminate the study at any time. All measurements were completed by all participants in the classroom under the standardized guidance of trained research assistants. Students provided their name and school number to be matched to the two waves of data.

### 3.4. Data Analysis

First, descriptive statistics and Pearson correlation analyses were performed in SPSS 26.0. Before conducting Pearson correlations, skewness and kurtosis were examined to evaluate the distributional assumptions of the study variables. Missing data were handled using full information maximum likelihood (FIML) within the SEM framework. Given that attrition was associated with an observed baseline variable, the data were considered unlikely to satisfy a strict MCAR assumption. However, the MAR assumption was treated as a plausible conditional on the observed variables included in the model. Given that all constructs were measured using adolescent self-reports, shared-method variance was a methodological concern. Thus, the findings should be interpreted as associations among adolescent-reported constructs, including perceived helicopter parenting and parent–child conflict. The two-wave design and autoregressive paths helped reduce, but could not eliminate, this concern. Second, to address our primary research question, analyses were conducted using structural equation modeling in Mplus 8.3. Configural, metric, and scalar invariance were tested longitudinally across T1 and T2 and across the high- and low-trait-autonomy groups. After measurement invariance was confirmed, observed composite scores for helicopter parenting, parent–child conflict, and short video addiction at T1 and T2 were created in Mplus using the DEFINE command and used as manifest variables in the cross-lagged, mediation, and multigroup models. A two-wave autoregressive cross-lagged model was used to examine the cross-lagged effect of helicopter parenting, parent–child conflict, and short video addiction. Gender, SES, parental education, and area were included as covariates. Third, after estimating the cross-lagged model, we further examined the indirect effect of helicopter parenting at T1 on short video addiction at T2 through parent–child conflict. Following Cole and Maxwell’s (2003) longitudinal mediation approach for two-wave panel data, the path from *X_T_*_1_ to *M_T_*_2_ can be set as “*a*,” the path from *M_T_*_1_ to *Y_T_*_2_ can be set as “*b*,” and the product a*b represents the mediation effect of *X* on *Y*. The indirect effect was tested using 5000 bootstrap samples and 95% confidence intervals. Finally, to examine differences by trait autonomy, we conducted a multiple-group analysis in which the path coefficients were constrained to be equal across groups. Participants were classified based on their trait autonomy scores at T1. Those scoring above the T1 sample mean were classified as the high-trait-autonomy group, whereas those scoring below the mean were classified as the low-trait-autonomy group. This resulted in 535 participants in the low-trait-autonomy group and 455 in the high-trait-autonomy group.

## 4. Results

### 4.1. Descriptive Statistics

Table 2 presents descriptive statistics, Pearson correlations, and normality diagnostics for helicopter parenting, parent–child conflict, and short video addiction at T1 and T2. Skewness values ranged from 0.17 to 1.46, and kurtosis values ranged from −0.26 to 3.84, indicating no severe departures from normality. Helicopter parenting was positively associated with parent–child conflict and short video addiction at two time points. Parent–child conflict was positively associated with short video addiction at two time points. Paired-samples *t* tests further showed significant increases from T1 to T2 in helicopter parenting, *t* = 3.31, *p* = 0.001, *dz* = 0.12, parent–child conflict, *t* = 2.42, *p* = 0.016, *dz* = 0.08, and short video addiction, *t* = 7.55, *p* < 0.001, *dz* = 0.26. Although statistically significant, these mean changes were small in magnitude.

### 4.2. Measurement Invariance

Measurement invariance was tested in two steps (Table 3). First, longitudinal measurement invariance was examined for helicopter parenting, parent–child conflict, and short video addiction across T1 and T2. The results supported configural, metric, and scalar invariance for all three constructs, indicating that they were measured in a sufficiently comparable manner across the two time points. Second, group measurement invariance was examined for trait autonomy before conducting the multigroup analysis. The results supported configural, metric, and scalar invariance across the high- and low-trait-autonomy groups, indicating that the trait autonomy measure functioned comparably across groups.

### 4.3. The Cross-Lagged Model

**Figure 1:**
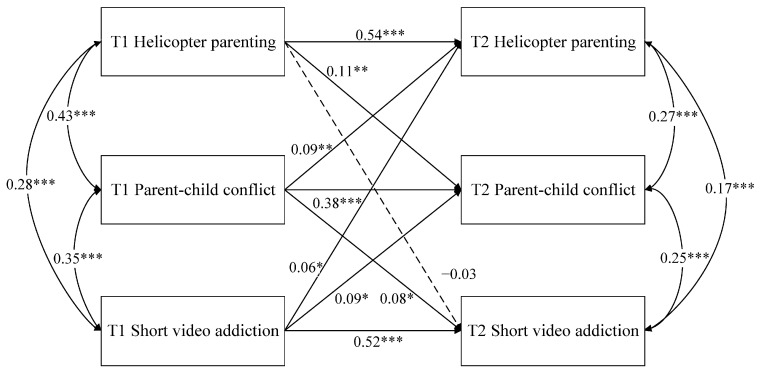
Cross-lagged Associations among helicopter parenting, parent–child conflict, and short video addiction across two waves. Standardized path coefficients are presented. Single-headed arrows indicate autoregressive and cross-lagged paths, curved double-headed arrows indicate within-time correlations, and dashed arrows indicate nonsignificant paths. * *p* < 0.05, ** *p* < 0.01, *** *p* < 0.001.

The cross-lagged panel model was constructed to investigate relations among helicopter parenting, parent–child conflict, and short video addiction (see Figure 1). In this model, the three T1 variables were freely correlated, and residual correlations among the three T2 variables were freely estimated to account for concurrent associations among the endogenous variables. Residual correlations across time were not specified. Given that the two-wave cross-lagged model was fully specified, it was saturated (*df* = 0). In saturated models, global fit indices are not substantively informative, as the model reproduces the observed covariance matrix perfectly by definition. Therefore, interpretation focused on the parameter estimates, including the autoregressive and cross-lagged paths (Steeger & Gondoli, 2013). The results showed that within-time correlations between helicopter parenting and parent–child conflict were significant at two time points (*r_T_*_1_ = 0.43, *p* < 0.001, *r_T_*_2_ = 0.27, *p* < 0.001). Correlations between helicopter parenting and short video addiction were significant at two time points (*r_T_*_1_ = 0.28, *p* < 0.001, *r_T_*_2_ = 0.17, *p* < 0.001). Correlations between parent–child conflict and short video addiction were significant at two time points (*r_T_*_1_ = 0.35, *p* < 0.001, *r_T_*_2_ = 0.25, *p* < 0.001). Moreover, autoregressive paths were significant for helicopter parenting, parent–child conflict, and short video addiction across time. In terms of cross-lagged effects, helicopter parenting at T1 did not predict short video addiction at T2 (*β* = −0.03, *p* = 0.42), but short video addiction at T1 positively predicted helicopter parenting at T2 (*β* = 0.06, *p* = 0.04). Helicopter parenting significantly positively predicted subsequent parent–child conflict (*β* = 0.11, *p* = 0.004). Reciprocally, parent–child conflict also significantly positively predicted subsequent helicopter parenting (*β* = 0.09, *p* = 0.006). Furthermore, parent–child conflict positively predicted short video addiction across time (*β* = 0.08, *p* = 0.04), and short video addiction also significantly and positively predicted parent–child conflict across time (*β* = 0.09, *p* = 0.01). Additionally, although several cross-lagged paths reached statistical significance, the standardized coefficients were modest in magnitude, ranging from 0.06 to 0.11. Recent guidelines for cross-lagged panel models suggest that effects of 0.03, 0.07, and 0.12 may be interpreted as small, medium, and large, respectively (Orth et al., 2024). Thus, the present findings should be interpreted as modest cross-lagged associations rather than strong predictive effects.

### 4.4. The Mediation Model and Multiple Group Analysis

We further tested the indirect effect of helicopter parenting at T1 on short video addiction at T2 through parent–child conflict. Following the two-wave longitudinal mediation approach proposed by Cole and Maxwell (2003), the indirect effect was estimated as the product of the path from helicopter parenting at T1 to parent–child conflict at T2 and the path from parent–child conflict at T1 to short video addiction at T2. Helicopter parenting at T1 positively predicted parent–child conflict at T2, *β* = 0.11, *p* = 0.004, and parent–child conflict at T1 positively predicted short video addiction at T2, *β* = 0.08, *p* = 0.04. The standardized indirect effect was statistically significant but very small, *β* = 0.012, 95% CI [0.002, 0.031]. However, the direct path from helicopter parenting at T1 to short video addiction at T2 was nonsignificant, *β* = −0.03, *p* = 0.42. Therefore, this finding suggests a small indirect effect of helicopter parenting at T1 on short video addiction at T2 through parent–child conflict.

**Figure 2:**
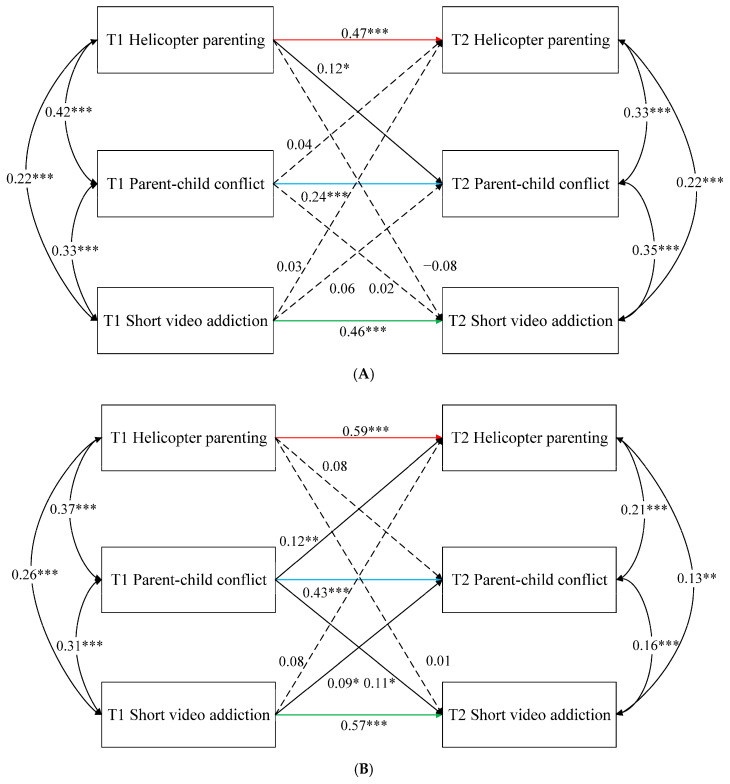
Cross-lagged associations among helicopter parenting, parent–child conflict, and short video addiction for high trait autonomy and low trait autonomy across two waves. (**A**) High-trait-autonomy group. (**B**) Low-trait-autonomy group. Note. Standardized path coefficients are presented. Significant group differences were found only for the three autoregressive paths; none of the cross-lagged paths differed significantly between groups. Single-headed arrows indicate autoregressive and cross-lagged paths, curved double-headed arrows indicate within-time correlations, and dashed arrows indicate nonsignificant paths. Colored paths indicate autoregressive paths with significant group differences. * *p* < 0.05, ** *p* < 0.01, *** *p* < 0.001.

A separate multigroup model was fit to evaluate trait autonomy differences. When the path coefficients across autonomy groups were restricted to be equal, the constrained model significantly differed from unconstrained model, Δ*χ*^2^(9) = 24.37, *p* = 0.003, suggesting that some path coefficients differed between the high- and low-trait-autonomy groups. Specifically, significant differences were obtained for three autoregressive paths (see Figure 2A,B): (a) the path from helicopter parenting at T1 to T2 (Δ*χ*^2^(1) = 4.65, *p* = 0.03; *β*_high_ = 0.47, *p* < 0.001 versus *β*_low_ = 0.59, *p* < 0.001), (b) the path from parent–child conflict at T1 to T2 (Δ*χ*^2^(1) = 6.71, *p* = 0.009; *β*_high_ = 0.24, *p* < 0.001 versus *β*_low_ = 0.43, *p* < 0.001), and (c) the path from short video addiction at T1 to T2 (Δ*χ*^2^(1) = 5.12, *p* = 0.02; *β*_high_ = 0.46, *p* < 0.001, *β*_low_ = 0.57, *p* < 0.001). Notably, significant group differences emerged only for the three autoregressive paths, whereas none of the cross-lagged paths differed significantly between the high- and low-trait-autonomy groups.

## 5. Discussion

The current study used a cross-lagged model to examine associations among helicopter parenting, parent–child conflict, and short video addiction, and further explored the moderating role of trait autonomy. The results suggested that helicopter parenting was not a significant prospective predictor of adolescents’ short video addiction, whereas short video addiction was positively associated with later helicopter parenting. There were reciprocal associations between helicopter parenting and parent–child conflict, as well as between parent–child conflict and adolescents’ short video addiction. Additionally, parent–child conflict showed a small indirect association between helicopter parenting at T1 and short video addiction at T2, and trait autonomy moderated the temporal stability of the three study variables.

### 5.1. Helicopter Parenting and Short Video Addiction

The findings partially supported Hypothesis 1a, showing that helicopter parenting at T1 did not significantly predict short video addiction at T2, whereas short video addiction at T1 positively predicted helicopter parenting at T2. Specifically, first, helicopter parenting did not predict adolescents’ short video addiction. One possible explanation is that, as adolescents enter the individuation-separation stage, the influence of helicopter parenting on adolescents’ maladaptive outcomes, such as short video addiction, may gradually weaken. Another possible explanation is that the present study used only a three-month interval. As a relatively stable and slowly evolving parenting pattern, helicopter parenting may require longer time intervals and more waves of measurement to detect its effect on short video addiction.

Second, adolescents’ short video addiction predicted subsequent helicopter parenting. One possible explanation is that short video addiction, as a problematic behavior, may elicit parents’ reactive restrictions, thereby increasing adolescents’ perceived levels of helicopter parenting. This finding is also consistent with the parenting literature suggesting that adolescents’ behavioral responses influence subsequent parenting practices (Koning et al., 2018). Together, these results offer initial evidence that may help guide future research on how helicopter parenting is associated with adolescents’ short video addiction.

### 5.2. Helicopter Parenting and Parent–Child Conflict

Consistent with Hypothesis 1b, the results showed reciprocal associations between helicopter parenting and parent–child conflict, suggesting that these two adolescent-reported family experiences are prospectively linked over time. Given that both helicopter parenting and parent–child conflict were reported by adolescents in the present study, the observed reciprocal associations may partly reflect shared adolescent perceptions. That is, adolescents who experience more negative emotions or hold more negative views of the parent–child relationship are more likely to report both higher parent–child conflict and greater helicopter parenting.

Specifically, first, helicopter parenting at T1 positively predicted parent–child conflict at T2. This finding is consistent with previous research, suggesting that helicopter parenting increases the likelihood of parent–child conflict (Leung, 2021; Segrin et al., 2012). One possible explanation is that helicopter parenting, characterized by intrusiveness, is likely to provoke adolescent resistance and associated parent–child conflict. Another possible explanation is that helicopter parenting may restrict adolescents’ need for independence, thereby increasing the likelihood of parent–child conflict. Second, parent–child conflict at T1 positively predicted helicopter parenting at T2. One possible explanation is that parent–child conflict may provoke parental control behaviors such as helicopter parenting, in an effort to dampen conflict.

### 5.3. Parent–Child Conflict and Short Video Addiction

Consistent with Hypothesis 1c, parent–child conflict and adolescents’ short video addiction were reciprocally associated. Specifically, parent–child conflict positively predicted adolescents’ later short video addiction, and short video addiction also positively predicted adolescents’ later parent–child conflict. These findings are consistent with the transactional model of development, suggesting that maladaptive family relationships and adolescents’ problem behaviors may influence each other (Sameroff, 2009; Steeger & Gondoli, 2013).

On the one hand, adolescents who experience higher levels of parent–child conflict are more likely to seek temporary escape or compensatory gratification through short videos. Conflictual family relationships may undermine adolescents’ social and psychological needs, making short videos an appealing way to distance themselves from conflict. On the other hand, short video addiction may intensify disagreements between parents and adolescents over media use, thereby increasing later parent–child conflict. Adolescents with higher levels of short video addiction may experience higher parental restriction, which they may perceive as a threat to their autonomy, and respond with resistance. Taken together, although the effect sizes observed in the present study were small, these findings provide preliminary evidence for the association between parent–child conflict and adolescents’ short video addiction.

### 5.4. The Indirect Effect of Parent–Child Conflict

Consistent with Hypothesis 2, the findings showed a statistically significant but small indirect association from helicopter parenting at T1 to short video addiction at T2 through parent–child conflict. This small indirect association can be understood within the broader developmental consequences of helicopter parenting. Helicopter parenting has been linked to poorer adjustment, including lower self-regulation, lower self-efficacy, and higher internalizing symptoms, and these associations appear to extend across multiple domains of adaptation (La Rosa et al., 2025; McCoy et al., 2024). Within this context, parent–child conflict may represent one relational pathway through which helicopter parenting is associated with adolescents’ short video addiction. However, the two-wave design limits strong conclusions about longitudinal mediation. Thus, this finding should be interpreted as preliminary evidence consistent with parent–child conflict serving as a limited intervening pathway.

### 5.5. The Moderating Role of Trait Autonomy

Hypothesis 3 was supported. Trait autonomy moderated the autoregressive paths of helicopter parenting, parent–child conflict, and short video addiction from T1 to T2. These findings are consistent with self-determination theory, suggesting that individuals high in trait autonomy have a strong and stable tendency to act on their own volition, values, and beliefs, and are capable of overcoming controlling elements in family environments (Jiao & Segrin, 2023; Ryan & Deci, 2017; Weinstein et al., 2012).

Specifically, all three variables showed greater stability among adolescents with lower trait autonomy than among those with higher trait autonomy. One possible explanation is that adolescents with higher trait autonomy may be better able to cope with stressful family experiences, such as helicopter parenting, making helicopter parenting less persistent. Similarly, adolescents with higher trait autonomy may respond more flexibly to conflict, making persistent patterns of parent–child conflict less likely. Furthermore, compared with low trait autonomy, high trait autonomy may also be associated with better regulation of short video use, thereby making short video addiction less likely to remain stable.

### 5.6. Limitations, Contributions, and Further Directions

Several limitations are worth noting. First, although the current study used a cross-lagged design to examine associations among the study variables, the traditional two-wave CLPM has important methodological limitations. Given that the model was saturated with zero degrees of freedom, global model fit could not be meaningfully evaluated. This constraint limits the extent to which the cross-lagged coefficients can be interpreted as evidence supporting a specific developmental process. In addition, the two-wave design prevented us from distinguishing within-person fluctuations from stable between-person differences. The three-month interval may be appropriate for detecting short-term directional associations involving parent–child conflict and short video addiction, but it may be relatively short for capturing changes in helicopter parenting, which is generally conceptualized as a more stable and slowly evolving parenting pattern. Future studies should use three or more waves and models such as the random-intercept CLPM to distinguish within-person dynamics from between-person associations more rigorously. Second, all variables were reported by adolescents, which may have introduced shared-method variance and inflated associations among the study variables. Although adolescent perceptions are important for understanding how parenting and family relationships are experienced, the absence of parent-reported data is a substantive limitation, especially for the assessment of helicopter parenting. Adolescents’ reports may reflect their subjective perceptions of parental behavior rather than parents’ actual intentions or practices. Thus, the observed associations involving helicopter parenting should be interpreted as associations based on adolescents’ perceived helicopter parenting. Future studies should include parent-reported and multi-informant data to reduce shared-method bias and provide a more comprehensive assessment of parenting processes. Third, short video addiction was measured using an adapted internet addiction scale. Although this approach has been used in prior research, it may not fully capture the platform-specific features of short video use. Future studies should employ dedicated short video addiction measures with stronger construct specificity. Fourth, attrition should be acknowledged as a limitation. Adolescents who dropped out after T1 reported higher levels of parent–child conflict at baseline, suggesting that adolescents with higher conflict levels were underrepresented at T2. As a result, the cross-lagged estimates involving parent–child conflict may have been attenuated or otherwise biased. Nevertheless, missing data were handled using FIML within the SEM framework, and the overall pattern of findings should still be interpreted with caution.

Despite limitations, the current study has several important implications. From a theoretical perspective, the present study adds to the literature by examining temporal sequences among helicopter parenting, parent–child conflict, and short video addiction. On this basis, the present study explores the indirect effect of parent–child conflict and the moderating role of trait autonomy. These findings provide preliminary evidence for the association between parent–child conflict and short video addiction. Second, the current study extends research related to helicopter parenting and adolescents’ addictive behaviors to the context of short video use. According to the transactional model of development and from a family systems perspective, the present study offers additional evidence regarding family-related factors that may contribute to adolescents’ short video addiction (Sameroff, 2009).

From a practical perspective, first, helicopter parenting was not a significant prospective predictor of short video addiction, whereas short video addiction was associated with later helicopter parenting. This finding suggests that practitioners should focus on intervening in the issue of adolescents’ short video addiction. Second, there are reciprocal associations between helicopter parenting and parent–child conflict. This finding indicates that intervention strategies need to consider the short-term effects of helicopter parenting, especially in the area of relationships. Intervention programs should also focus on providing parents and adolescents with information aimed at resolving conflicts. Third, the findings suggest reciprocal associations between parent–child conflict and short video addiction. Thus, educators should consider both adolescents’ short video addiction and parent–child conflict when designing intervention programs. These findings also suggest that efforts to reduce adolescents’ short video addiction may benefit from involving both adolescents and their parents. Fourth, adolescents with low trait autonomy are more likely to be affected by family pressure and engage in inappropriate behaviors. Practitioners may also help adolescents with low trait autonomy develop more adaptive self-regulation skills in family contexts.

## Figures and Tables

**Table 1:** Demographic information of the sample.

Demographic Information	Level	Percentage
Gender	Males	46.7
	Females	52.7
	Not reported	0.6
Fathers’ education	Elementary or lower	9.7
	Junior middle school	35.5
	Senior middle school	25.7
	Bachelor’s degree or higher	29.0
Mothers’ education	Elementary or lower	12.1
	Junior middle school	36.9
	Senior middle school	23.2
	Bachelor’s degree or higher	27.9
Per capita monthly family income	<2000	7.3
	2000–4000	15.2
	4000–6000	23.5
	6000–10,000	30.1
	>10,000	23.9

**Table 2:** Descriptive statistics and Pearson correlations among variables of interest.

	1	2	3	4	5	6
1. T1 Helicopter parenting	—					
2. T2 Helicopter parenting	0.60 ***	—				
3. T1 Parent–child conflict	0.43 ***	0.35 ***	—			
4. T2 Parent–child conflict	0.29 ***	0.40 ***	0.45 ***	—		
5. T1 Short video addiction	0.27 ***	0.24 ***	0.35 ***	0.24 ***	—	
6. T2 Short video addiction	0.14 ***	0.24 ***	0.24 ***	0.34 ***	0.54 ***	—
M	2.51	2.58	5.18	5.51	2.38	2.59
SD	0.61	0.61	3.71	3.82	0.82	0.84
Skewness	0.26	0.16	1.46	1.43	0.28	0.25
Kurtosis	0.11	−0.05	3.04	3.84	−0.25	0.26

Note. T1 = Time 1; T2 = Time 2. *** *p* < 0.001.

**Table 3:** Tests of longitudinal and group measurement invariance.

Panel	Construct	Model	*χ*^2^(*df*)	CFI	TLI	RMSEA (90% CI)	SRMR	∆CFI	∆TLI	∆RMSEA
Panel A. Longitudinal invariance	Helicopter parenting	Configural	179.897 (63)	0.953	0.932	0.048 [0.040, 0.056]	0.049	—	—	—
		Metric	190.521 (69)	0.951	0.935	0.046 [0.039, 0.054]	0.050	0.002	0.002	0.001
		Scalar	200.069 (74)	0.949	0.937	0.046 [0.038, 0.053]	0.050	0.002	0.000	0.000
	Parent–child conflict	Configural	162.897 (91)	0.984	0.979	0.031 [0.023, 0.039]	0.034	—	—	—
		Metric	177.115 (98)	0.983	0.979	0.031 [0.024, 0.039]	0.037	0.001	0.000	0.003
		Scalar	186.687 (104)	0.982	0.979	0.031 [0.024, 0.038]	0.037	0.001	0.000	0.000
	Short video addiction	Configural	300.472 (87)	0.952	0.934	0.055 [0.048, 0.062]	0.041	—	—	—
		Metric	316.963 (94)	0.950	0.937	0.054 [0.047, 0.061]	0.043	0.002	0.003	0.001
		Scalar	337.326 (99)	0.947	0.936	0.054 [0.048, 0.061]	0.044	0.003	0.001	0.000
Panel B. Group invariance	Trait autonomy	Configural	318.718 (95)	0.928	0.909	0.054 [0.047, 0.060]	0.045	—	—	—
		Metric	349.292 (102)	0.920	0.906	0.055 [0.048, 0.061]	0.058	0.008	0.003	0.001
		Scalar	376.593 (109)	0.914	0.905	0.055 [0.049, 0.061]	0.056	0.006	0.001	0.000

Note. CFI = Comparative Fit Index; RMSEA = Root Mean Square Error of Approximation; CI = Confidence Interval.

## Data Availability

The datasets generated and/or analyzed during the current study are not publicly available but are available from the corresponding author on reasonable request. This study was not pre-registered.
